# A phase I trial of cytotoxic T-lymphocyte precursor-oriented peptide vaccines for colorectal carcinoma patients

**DOI:** 10.1038/sj.bjc.6601711

**Published:** 2004-03-02

**Authors:** Y Sato, Y Maeda, H Shomura, T Sasatomi, M Takahashi, Y Une, M Kondo, T Shinohara, N Hida, K Katagiri, K Sato, M Sato, A Yamada, H Yamana, M Harada, K Itoh, S Todo

**Affiliations:** 1 First Department of Surgery, Hokkaido University School of Medicine, Sapporo, Japan; 2 Department of Immunology, Kurume University School of Medicine, Fukuoka, Japan; 3 Department of Surgery, Kurume University School of Medicine, Fukuoka, Japan

**Keywords:** peptide, cancer vaccine, immunotherapy, colon cancer, CTL

## Abstract

In most protocols of peptide-based vaccination, no consideration has been paid to whether or not peptide-specific cytotoxic T-lymphocyte (CTL) precursors are pre-existent in cancer patients. Initiation of immune boosting through vaccination is better than that of immune priming to induce prompt and strong immunity. In this study, 10 human histocompatibility leukocyte antigen-A24^+^ patients with advanced colorectal carcinomas were treated with up to four peptides that had been positive for pre-vaccination measurement of peptide-specific CTL precursors in the circulation (CTL precursor-oriented peptide vaccine). No severe adverse effect was observed, although local pain and fever of grade I or II were observed. Post-vaccination peripheral blood mononuclear cells (PBMCs) from five patients demonstrated an increased peptide-specific immune response to the peptides. Increased CTL response to cancer cells was detected in post-vaccination PBMCs of five patients. Antipeptide immunoglobulin G became detectable in post-vaccination sera of seven patients. Three patients developed a positive delayed-type hypersensitivity response to at least one of the peptides administrated. One patient was found to have a partial response; another had a stable disease, sustained through 6 months. These results encourage further development of CTL precursor-oriented vaccine for colorectal cancer patients.

Recent advances in molecular biology and cellular immunology have resulted in identification of various antigens and epitopes recognised by human histocompatibility leukocyte antigen (HLA)-class-I-restricted cytotoxic T lymphocytes (CTLs) from various cancers (Bruggen *et al*, 1991; Brichard *et al*, 1993; Kawakami *et al*, 1994a, 1994b Fisk *et al*, 1995; Peoples *et al*, 1995; Robbins *et al*, 1996; Correale *et al*, 1997; Correale *et al*, 1998). Many clinical trials of peptide-based immunotherapy have shown an increased immune response to the vaccinated peptides (Rosenberg *et al*, 1998; Marchand *et al*, 1999; Jager *et al*, 2000; Gajewski *et al*, 2001; Gjertsen *et al*, 2001; Lau *et al*, 2001; Valmori *et al*, 2001). However, these initial clinical studies have produced few clinical responses in the various types of cancer treated including melanoma and colorectal cancer (Finn and Lotze, 2001; Miyagi *et al*, 2001; Sadanaga *et al*, 2001). This failure could be in part due to the insufficient and late stages of CTL induction by the current regimen, in which pre-vaccination screening of suitable peptides for each patient among a large number of candidate peptides is not conducted. As a result, vaccination in the present study began with immune priming. This standard regimen could be effective in preventing infectious disease. However, the main goal of a cancer vaccine is treatment of malignant disease. The time-lag necessary for priming of an antitumour response should be seriously considered, as the expected survival of most advanced cancer patients under these regimens is 3–9 months (Cole and Rodu, 2001; Miyagi *et al*, 2001). Therefore, the development of a new regimen of therapeutic vaccine is needed (Finn and Lotze, 2001). One regimen might include pre-vaccination measurement of peptide-specific CTL precursors in the circulation, followed by vaccination of only CTL-reactive peptides (CTL precursor-oriented vaccine). We have previously reported 14 vaccine candidate peptides that can induce HLA-A24-restriced and tumour-specific CTL in cancer patients (Gomi *et al*, 1999; Kikuchi *et al*, 1999; Yang *et al*, 1999; Harashima *et al*, 2000; Kawano *et al*, 2000; Nakao *et al*, 2000; Nishizaka *et al*, 2000). We have also shown that most cancer patients have peptide-specific CTL precursors for some of these peptides, and that peripheral blood mononuclear cells (PBMCs) stimulated with positive peptides show HLA-class-I-restricted and tumour-specific cytotoxicity (Hida *et al*, 2002; Maeda *et al*, 2002; Suzuki *et al*, 2002). In the present study, patients with advanced stages of colorectal cancer were immunised with up to four peptides identified in pre-vaccination measurement of peptide-specific CTL precursors to evaluate the toxicities and responses to CTL precursor-oriented peptide vaccination.

## MATERIALS AND METHODS

### Patients and eligibility criteria

The study protocol was approved by the Institutional Ethical Review Boards of Hokkaido University and Kurume University, respectively. Complete written informed consent was obtained from all of patients at the time of enrolment. According to the protocol, patients were required to be HLA-A24 positive, and to have a histologically confirmed lesion of colorectal carcinoma. Eligibility criteria included an age of 85 years or less, serum creatinine of less than 1.4 mg dl^−1^, bilirubin of less than 1.5 mg dl^−1^, a platelet count of 100 000 *μ*l^−1^ or more, haemoglobin of 8.0 g dl^−1^ or more, and total WBC of 3000 *μ*l^−1^ or more. Hepatitis B surface antigen, Hepatitis C antigen, and human immunodeficiency virus (HIV) were required to be negative. The patients, who had been untreated for at least 4 weeks before the study, had an Eastern Cooperative Oncology Group performance status of 0–2. The treatment was carried out at the First Department of Surgery, Hokkaido University School of Medicine (patients 1–9) and the Department of Surgery, Kurume University School of Medicine (patient 10) from November 2000 through April 2002. All immunological analyses were carried out at the Department of Immunology, Kurume University School of Medicine.

### Screening of peptide-specific CTL precursors

A volume of 30 ml of peripheral blood was obtained pre- and post- (3rd, 6th, and 9th) vaccination, and PBMCs were isolated by means of Ficoll–Conray density gradient centrifugation, as reported previously (Miyagi *et al*, 2001). A previously reported method was used to detect peptide-specific CTL precursors in PBMCs (Hida *et al*, 2002; Suzuki *et al*, 2002). Briefly, PBMCs (1 × 10^5^ cells well^−1^) were incubated with 10 *μ*M of a peptide in wells of u-bottom-type 96-well microculture plates (Nunc, Roskilde, Denmark) in 200 *μ*l of culture medium. The culture medium consisted of 45% RPMI-1640 medium, 45% AIM-V® medium (GIBCO BRL), 10% FCS, 100 U ml^−1^ of interleukin (IL)-2, and 0.1 *μ*M MEM nonessential amino-acid solution (GIBCO BRL). Every 3 days, half of the medium was removed and replaced with new medium containing a corresponding peptide (20 *μ*M). The assay was performed in quadruplicate. After a 12-day incubation, the cultured cells in one well were divided into four wells, two of which were used for corresponding peptide-pulsed C1R-A2402 cells, and the other two of which were used for HIV peptide (RYLRQQLLGI)-pulsed C1R-A2402 cells. The HLA-A24-binding HIV peptide was used as a negative control. After an 18-h incubation period, the supernatants were collected and the level of interferon (IFN)-*γ* was determined by enzyme-linked immunosorbent assay (ELISA) (limit of sensitivity: 10 pg ml^−1^). The background response to HIV peptide-pulsed C1R-A2402 cells was subtracted from the value. Assessment of peptide-specific CTL precursors was performed based on two parameters, the *P*-value by the Student's *t*-test and IFN-*γ* production, as described in legends of Tables 2 and 4. According to the results of this test, up to four positive peptides were selected for each patient, utilised in a skin test, and then, if negative, injected as a vaccination. The screening of peptide-specific CTL precursors was performed by the same method after the 6th vaccination to evaluate the effects of immunisation.

### Peptides and vaccination

The peptides utilised in the present study were prepared under conditions of Good Manufacturing Practice using a Multiple Peptide System (San Diego, CA, USA). Montanide ISA-51, an incomplete adjuvant, was manufactured by Seppic, Inc (Franklin Lakes, NJ, USA). The peptides were supplied in vials containing 3 mg ml^−1^ sterile solution for injection. A 3 mg portion of peptide with sterile saline was added in a 1 : 1 volume to Montanide ISA-51, then mixed in a Vortex mixer (Fisher, Inc, Alameda, CA, USA). The resulting emulsion was injected subcutaneously into the lateral thigh using a glass syringe. Patients were vaccinated every 14 days for a total of three injections to measure the toxicity. For the patients with no toxicity, the vaccinations were repeated biweekly up to 15 times with informed consent from each patient.

### Delayed-type hypersensitivity (DTH) skin test

Skin tests were performed using 50 *μ*g of each peptide injected intradermally in a volume of 100 *μ*l using a tuberculin syringe and a 27-gauge needle. Saline was a negative control for assessment of DTH. At least 7 mm of induration or erythema read 48 h after injection was needed to score the skin test as positive (Nestle *et al*, 1998).

### Assay of cytotoxicity

Cytotoxic activity was measured using a standard 6-h ^51^Cr-release assay (Gomi *et al*, 1999; Miyagi *et al*, 2001). Cryopreserved PBMCs were thawed and cultured in the medium consisting of 45% RPMI-1640 medium, 45% AIM-V® medium (GIBCO BRL), 10% FCS, 100 U ml^−1^ of IL-2, and 0.1 *μ*M MEM nonessential amino-acid solution (GIBCO BRL). On the 14th day of culture, the cells were harvested and served for the assay. To avoid the bias of bioassays, PBMCs harvested at different times from a single patient were thawed at the same time. SW620 (HLA-A24^+^, colon adenocarcinoma), COLO201 (HLA-A24^−^, colon adenocarcinoma), and PHA-blastoid T cells (HLA-A24^+^) were used as target cells (1 × 10^3^ well^−1^), and 50-fold unlabelled K562 cells were added into wells to decrease nonspecific killing activity.

### Purification of CD8^+^ or CD4^+^ T cells

CD8^+^ or CD4^+^ T cells were positively isolated from peptide-stimulated PBMCs using the CD8 Positive Isolation Kit (DYNAL, Oslo, Norway) or the CD4 Positive Isolation Kit (DYNAL) according to the manufacturer's instructions. In both cases, the percentage of purified CD8^+^ or CD4^+^ T cells was more than 90% (data not shown).

### Kinetics of peptide-specific CTL precursors

To detect the kinetics of peptide-specific CTL precursor frequency in patient 1, PBMCs from before and after vaccination were incubated at 100 cells per well of a 96-well u-bottom microculture plate in the presence of feeder cells without the peptides. Cells from each well were harvested on the 14th day of culture and tested for their ability to produce IFN-*γ* by recognition of peptide-pulsed CIR-A2402 cells in duplicate assay. The well was considered positive if it contained effector cells producing much higher than 100 pg ml^−1^ and also statistically significant levels (*P*<0.05 by the Student's *t*-test) of IFN-*γ* in response to CIR-A2402 cells preloaded with a corresponding peptide as compared with those in response to the HIV peptide-pulsed CIR-A2402 cells.

### Detection of serum immunoglobulin G (IgG) levels

An ELISA was used to detect the serum IgG levels specific to the peptides administered, as reported previously (Miyagi *et al*, 2001). Briefly, the peptide (20 *μ*g well^−1^)-immobilised plate was blocked with Block Ace (Yukijirushi, Tokyo, Japan), washed, and 100 *μ*l well^−1^ of serum samples diluted with 0.05% Tween 20-Block Ace were added to the plate. After a 2-h incubation, the plate was washed and further incubated for 2 h with an 1 : 1000-diluted rabbit anti-human IgG (DAKO, Glostrup, Denmark). The plate was washed, after which 100 *μ*l of 1 : 100-diluted goat anti-rabbit Ig-conjugated horseradish peroxidase-dextran polymer (En Vision, DAKO) was added to each well, and the plate was incubated for 40 min. After washing, 100 *μ*l well^−1^ of tetramethyl-benzidine substrate solution (KPL, Guildford, UK) was added, and the reaction was stopped by the addition of 1 M phosphoric acid. To estimate peptide-specific IgG levels, the optical density values of each sample were compared with those of serially diluted standard samples, and the values are shown as the optical density. The specificity of the ELISA was tested as follows. Serum samples (1 : 100 diluted) were incubated in wells precoated with corresponding or irreverent peptides to absorb the peptide-specific IgG. After a 2-h incubation at room temperature, samples were transferred to new wells precoated with the sample peptide used in the first absorption, and this protocol was repeated for a total of three times. The samples were then subjected to peptide-specific IgG ELISA.

### Evaluation of treatment response

All known sites of disease were evaluated by CT-scan or X-ray examination pre- and post-vaccinations (the 3rd, 6th, 9th, and 12th). Patients were assigned a response category according to the response evaluation criteria in solid tumours, a revised version of the WHO criteria published in the WHO Handbook for reporting results of cancer treatment in June 1999.

## RESULTS

### Demographics of the patients

In all, 10 patients with advanced colorectal carcinomas were enroled in this phase I study. Demographic details of the patients are shown in Table 1

**Table 1:** Patient characteristics

**Patient**	**Age**	**Sex**	**Primary site**	**Metastasis site**	**Previous treatment^a^**	**P.S.^b^**	**No. of vaccination received^c^**
1	67	M	Transverse colon	Liver, para-aortic lymph node	S. C	0	15
2	38	F	Transverse colon	Liver, peritoneum (intrapelvic)	S. C	0	12
3	67	M	Ascending colon	Lung, liver	S. C	1	6
4	78	M	Rectum	Liver	S. C. M	0	10
5	70	F	Sigmoid colon	Lung, liver	S. C	1	11
6	72	M	Rectum	Liver	S	1	10
7	67	M	Sigmoid colon	Lung	S. C	0	8
8	44	M	Sigmoid colon	Lung, liver	S. C	0	6
9	46	M	Ascending colon	Peritoneum (intrapelvic)	S. C	0	6
10	28	F	Sigmoid colon	Peritoneum (intrapelvic)	S. C	1	8

a S = surgery; C = chemotherapy; M = percutaneous microwave coagulation therapy.

b P.S. = performance status by ECOG score.

c The initial protocol consisted of three vaccinations; additional vaccinations were subsequently performed to patients who showed a favourable clinical course after they provided further informed consent.

. The median patient age was 67 years (range 28–78). All patients had liver, lung, peritoneum, or lymph node metastases. All patients underwent surgical resection of the primary lesion; nine had failed previous chemotherapy. All 10 patients completed the first three vaccinations within the protocol under informed consent, and all of them received more vaccinations (6–15) under additional informed consent.

### Screening of peptide-specific CTL precursors

All tumour-related antigens were identified by screening of a cDNA library from tumour cells using tumour-reactive CTLs. The peptides used for this study have the potential to induce HLA-A24-restricted and tumour-specific CTL activity in PBMCs of HLA-A24^+^ cancer patients (Kikuchi *et al*, 1999; Yang *et al*, 1999; Gomi *et al*, 1999; Harashima *et al*, 2000; Kawano *et al*, 2000; Nakao *et al*, 2000; Nishizaka *et al*, 2000). Pre-vaccination PBMCs were provided for screening of the CTL precursors reactive to the 14 candidate peptides, followed by selection of peptides based on evaluation of the results with the criteria shown in Table 2

**Table 2:** Pre-vaccination screening of peptide-specific CTL precursors

		**Patients**											
**Peptide**	**Sequence**	**1**	**2**	**3**	**4**	**5**	**6**	**7**	**8**	**9**	**10**	**Positive case**	**Vaccinated case**
SART1 690	EYRGFTQDF								○A	○AA		2/10	2/10
SART2 93	DYSARWNEI								○AB			1/10	1/10
SART2 161	AYDFLYNYL								○A	○AA		2/10	2/10
SART2 899	SYTRLFLIL	•B										1/10	0/10
SART3 109	VYDYNCHVDL	○A	○B			○C	○C	○A		○A		6/10	6/10
SART3 315	AYIDFEMKI							○C			○C	2/10	2/10
CyB 84	KFHRVIKDF							•A				1/10	0/10
CyB 91	DFMIQGGDF			○A	○A			○C			○A	4/10	4/10
lck 208	HYTNASDGL	○C	○A		○A	○C		○C	○B		○AAA	7/10	7/10
lck 488	TFDYLRSVL		○AB		○AAAA					○AB	○A	4/10	4/10
lck 488	DYLRSVLEDF	○A		○A	○AA							3/10	3/10
ART1 170	EYCLKFTKL	•B			•A		•A			•AA		4/10	0/10
ART4 13	AFLRHAAL					•C						1/10	0/10
ART4 75	DYPSLSATDI			○A			○B					2/10	2/10

White circles indicate that the peptide was positive for the CTL precursor induction assay and was injected. Black circles indicate that the peptide was positive for the CTL precursor induction assay but was not administrated due to immediate-type hypersensitivity by skin test. The assay was performed in quadruplicate and the background response to the HIV peptide was subtracted from the value. The result was evaluated by the following criteria: A, *P*<0.05 and IFN-*γ* production >50 pg ml^−1^; B, *P*<0.05 and 50 pg ml^−1^ >IFN-*γ* production >25 pg ml^−1^: C, 0.05<*P*<0.1 and IFN-*γ* production >25 pg ml^−1^. The classification is shown by letters of the alphabet, and each character represents the results of each well. For example, ABC means that three wells were judged as A, B, and C, and one well was negative.

. The assay was performed in quadruplicate. After a 12-day incubation, the cultured cells in one well were separated into four wells, two of which were used for corresponding peptide-pulsed C1R-A2402 cells, and the other two of which were used for HIV peptide-pulsed C1R-A2402 cells. The HLA-A24-binding HIV peptide was used as a negative control. The assessment of peptide-specific CTL precursors was performed based on two parameters, the *P*-value by the Student's *t*-test and IFN-*γ* production, as shown in the table legend. When these peptides were found to induce immediate-type hypersensitivity by a skin test, a fifth peptide was vaccinated if it proved negative in the skin test. SART2_899_, CyB_91_, ART1_170_, and ART4_13_ were positive for immediate-type hypersensitivity in all patients tested and were not injected at all. As a result, five patients were injected with four peptides, three patients with three peptides, and two with two peptides. The vaccinated peptides for each patient are shown in Table 2. It is noteworthy that the profiles of the vaccinated peptides varied greatly among the 10 patients.

### Toxicities

All 10 patients were evaluated for toxicity; the overall toxicities are shown in Table 3

**Table 3:** Toxicities associated with the peptide vaccination

	**Grade^a^**		
**Toxicities**	**I**	**II**	**Total**
Anorexia	1		1
Dermatologic	7	1	8
Diarrhoea	1		1
Fatigue	2		2
Fever	2	2	4
Nausea	2		2
Vomiting	1		1

a Toxicities are based on the NIH Common Toxicity Criteria.

. The vaccinations were generally well-tolerated, but almost all patients (eight out of 10) had grade I or II local redness and swelling at the injection sites. Fever with mild flu-like symptoms was observed in four patients (grade I or grade II), although this symptom was transient and no medication was needed. Grade I fatigue or nausea was observed in two patients, and grade I anorexia, diarrhoea, or vomiting was observed in one. No vaccine-related grade III or IV toxicity was observed (data not shown). There was no clinical evidence of an autoimmune reaction as determined by symptoms, physical examination, or laboratory test.

### Cellular immune responses

Post-vaccination (6th) PBMCs showed increased amounts of peptide-specific IFN-*γ* production compared to pre-vaccination PBMCs in five out of 10 patients (1, 2, 5, 6, and 10), as described in Table 4

**Table 4:** Summary of response to the peptide vaccination

		**Peptide-specific CTL^a^**		**Ab to peptide**		**DTH**		**Clinical response/**
**Patient**	**Peptide**	**Pre**	**Post**	**Pre**	**Post^b^**	**Pre**	**Post^b^**	**Time to progression (months)**
1	SART3 109	A	C	−	−	−	7 mm (9)	PR/7<
	lck 208	C	ArArA	−	+ (6)	−	10 mm (6)	
	lck 488	A	Ar	−	+ (9)	−	10 mm (6)	
								
2	SART3 109	B	−	−	+ (6)	−	−	SD/7
	lck 208	A	AAAA	−	−	−	−	
	lck 486	AB	A	−	+ (9)	−	−	
								
3	CyB 91	A	C	−	−	−	−	PD/3
	lck 488	A	A	−	−	−	−	
	ART4 75	A	−	−	−	−	−	
								
4	CyB 91	A	−	−	−	−	−	PD/3
	lck 208	A	C	−	−	−	7 mm (3)	
	lck 486	AAAA	C	−	−	−	7 mm (3)	
	lck 488	AA	−	−	−	−	7 mm (3)	
								
5	SART3 109	C	ArB	−	−	−	−	PD/4
	lck 208	C	A	−	+ (9)	−	−	
								
6	SART3 109	C	ArBC	−	+ (6)	−	−	PD/3
	ART4 75	B	B	−	−	−	−	
								
7	SART3 109	A	A	−	+ (6)	−	10 mm (3)	PD/4
	SART3 315	C	−	−	−	−	−	
	CyB 91	C	−	−	−	−	−	
	lck 208	C	C	−	−	−	−	
								
8	SART1 690	A	−	−	−	−	−	PD/4
	SART2 93	AB	−	−	−	−	−	
	SART2 161	A	C	−	−	−	−	
	lck 208	B	−	−	−	−	−	
								
9	SART1 690	AA	−	−	−	−	−	PD/4
	SART2 161	AA	−	−	−	−	−	
	SART3 109	A	−	−	−	−	−	
	lck 486	AB	C	−	+(6)	−	−	
								
10	SART3 315	C	ArAA	−	+(6)	−	−	PD/3
	CyB 91	A	−	−	−	−	−	
	lck 208	AAA	−	−	−	−	−	
	lck 486	AA	−	−	−	−	−	

a The CTL precursor induction assay was performed in quadruplicate, and the background response to the HIV peptide was subtracted from the value. The result was evaluated by the following criteria: Ar (armed response), P<0.1 and IFN-*γ* production >500 pg ml^−1^; A, *P*<0.05 and IFN-*γ* production >50 pg ml^−1^: B, *P*<0.05 and 50 pg ml^−1^ >IFN-*γ* production >25 pg ml^−1^; C, 0.05<*P*<0.1 and IFN-*γ* production >25 pg ml^−1^. The classification is shown by letters of the alphabet, and each character represents the results of each well. For example, ArBC means that three wells were judged as Ar, B, and C, and one well was negative.

b The number in the parenthesis represents the vaccination when anti-peptide IgG or DTH was detected for the first time.

**Figure 1:**
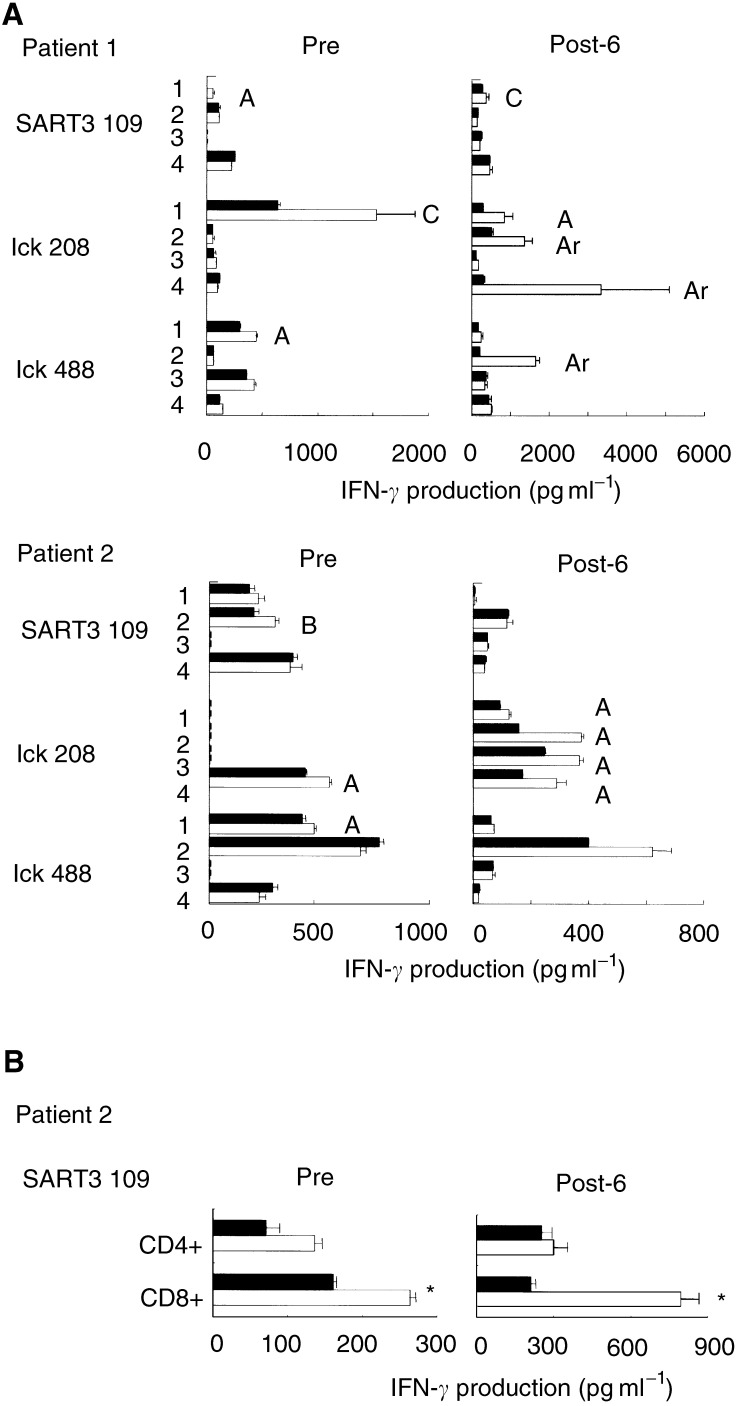
Assay of peptide-specific CTL precursors. (**A**) Pre- and post- (6th) vaccination PBMCs were provided for screening of reactivity to each of the 14 peptides listed in Table 2 in the quadruplicate assays. Representative results of patients 1 and 2 are shown in this figure. The peptide-stimulated PBMCs were cultured with C1R-A2402 cells that were preloaded with the corresponding peptide (open bar) or the HIV peptide (closed bar). The level of IFN-*γ* in the supernatant was determined by ELISA. The result was evaluated by the classification shown in the legend of Table 4. Each alphabet character represents the result of each well. (**B**) Pre- and post- (6th) vaccination PBMCs from patient 2 were stimulated *in vitro* with the SART3_109_ peptide. The peptide-stimulated PBMCs were harvested, and positively isolated CD4^+^ or CD8^+^ T cells were cultured in triplicate with C1R-A2402 cells that were preloaded with the SART3_109_ peptide (open bar) or the HIV peptide (closed bar). The level of IFN-*γ* in the supernatant was determined by ELISA. ^*^Statistically significant at *P*<0.05.

. Representative results of patients 1 and 2 are shown in Figure 1A

. In patients 1 and 2, CTL response to the lck_208_ was apparently induced after the 6th vaccination. In five other patients, peptide-specific CTL response decreased. We further tested the reactivity of purified CD4^+^ or CD8^+^ T cells in response to the administered peptides. The pre- or post-6th vaccination PBMCs from patient 2 were *in vitro* stimulated, and purified CD4^+^ or CD8^+^ T cells were tested for their reactivity to the SART3_109_ peptide-pulsed C1R-A2402 cells. As shown in Figure 1B, purified CD8^+^ T cells from the post-vaccination PBMCs of patient 2 produced IFN-*γ* in an antigen-specific manner, although no definite IFN-*γ* production specific to the SART3_109_ peptide was observed when unseparated post-6th PBMCs from patient 2 were used (Figure 1A). Purified CD4^+^ T cells failed to produce IFN-*γ* in a peptide-specific manner. On the other hand, no peptide-specific IL-4 production was observed in the case with purified CD8^+^ or CD4^+^ T cells (data not shown).

**Figure 2:**
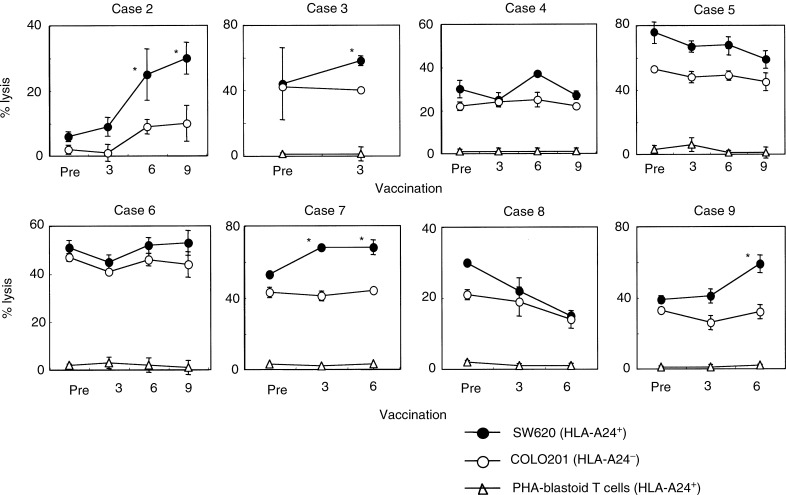
Cytotoxicity before and after the peptide vaccination. Pre- and post-vaccination PBMCs from eight patients were incubated for 14 days with IL-2 alone without any peptides in culture, followed by measurement of cytotoxicity against SW620 cells (HLA-A24^+^ colon cancer cell line), COLO201 cells (HLA-A24^−^ colon cancer cell line), and PHA-activated T cells (HLA-A24^+^) by a 6-h ^51^Cr-release assay at an *E*/*T* ratio of 40/1. The assay was performed in triplicate, and the mean and s.d. are shown. ^*^Statistically significant at *P*<0.05.

We next examined cytotoxicity of pre- and post- (3rd, 6th, and 9th) vaccination PBMCs from eight patients against SW620 (HLA-A24^+^ colon tumour cells), COLO201 cells (HLA-A24^−^ colon tumour cells), and PHA-activated T cells (HLA-A24^+^) (Figure 2

). Tumour-related antigens from which all peptides used in this study were derived are nonmutated self-antigens overexpressed in tumour cells, including SW620 and COLO201 (Shichijo *et al*, 1998; Yang *et al*, 1999; Gomi *et al*, 1999; Harashima *et al*, 2000; Kawano *et al*, 2000; Nakao *et al*, 2000; Nishizaka *et al*, 2000). As shown in four cases of patients 2, 3, 7, and 9, cytotoxicity against HLA-A24^+^ SW620 increased after peptide vaccination compared to that against HLA-A24^−^ COLO201. In the other four cases, no definite increase in cytotoxicity was observed after the peptide vaccination. No cytotoxicity against HLA-A24^+^ PHA-blastoid T cells was detected in any case. These results indicate that the peptide vaccination resulted in augmented CTL activity in four out of eight patients.

### Serum IgG specific to peptides

**Figure 3:**
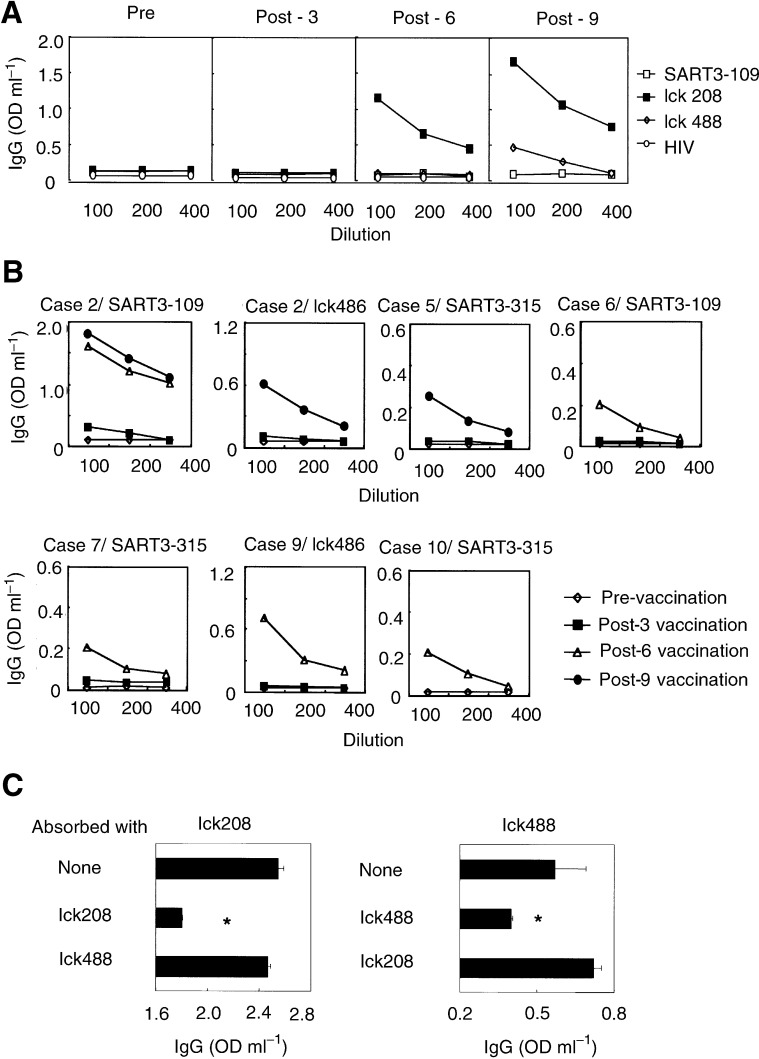
IgG reactive to the vaccinated peptides. (**A**) Pre- and post-vaccination sera from patient 1 were serially diluted and the levels of IgGs reactive to three administered peptides were determined by ELISA. (**B**) Pre- and post-vaccination sera from patients 2, 5, 6, 7, 9, and 10 were serially diluted and the levels of IgGs reactive to corresponding peptides were determined by ELISA. (**C**) Diluted sera of patient 1 after the 9th vaccination were cultured in the indicated peptide-coated wells and the levels of IgGs reactive to corresponding peptides were determined by ELISA. ^*^Statistically significant at *P*<0.05.

No IgG reactive to any of the vaccinated peptides was detected in pre-vaccination sera from any of the 10 patients (Table 4). Significant levels of anti-peptide IgG reactive to SART3- or lck-derived peptides became detectable in the post-vaccination sera of seven patients. Detail results are shown in Figure 3

. In patient 1, IgGs reactive to the lck_208_ and the lck_488_ peptides were induced after the 6th and 9th vaccinations, respectively (Figure 3A). A similar result was observed in six other patients (Figure 3B). It is noteworthy that three patients who showed strong peptide-specific CTL response (criteria Ar) after the peptide vaccination were also positive for antipeptide IgG to the corresponding peptides (patient 1 for lck_208_ and lck_488_, patient 6 for SART3_109_, and patient 10 for SART3_315_). In addition, the IgG response to the lck_208_ peptide in post-vaccination sera of patient 1 was neutralised by absorption with a corresponding peptide, but not with the lck_408_ peptide, whereas the opposite was observed in the case of the IgG response to the lck_488_ peptide (Figure 3C). This peptide-specific absorption demonstrates the validity of the ELISA system.

### DTH skin test

No DTH reaction against peptides was observed before vaccination in any patient, while peptide-specific DTH reactions were observed in three patients after the peptide vaccination (Table 4). In patient 1, DTH reactions to lck_208_ and lck_488_ were observed after the 6th vaccination, and DTH against SART3_109_ became detectable after the 9th vaccination. Patient 4 exhibited a DTH reaction against lck_208_, lck_486_, and lck_488_ after the 3rd vaccination. Patient 7 exhibited a DTH reaction against SART3_109_ after the 3rd vaccination.

### Clinical results

**Figure 4:**
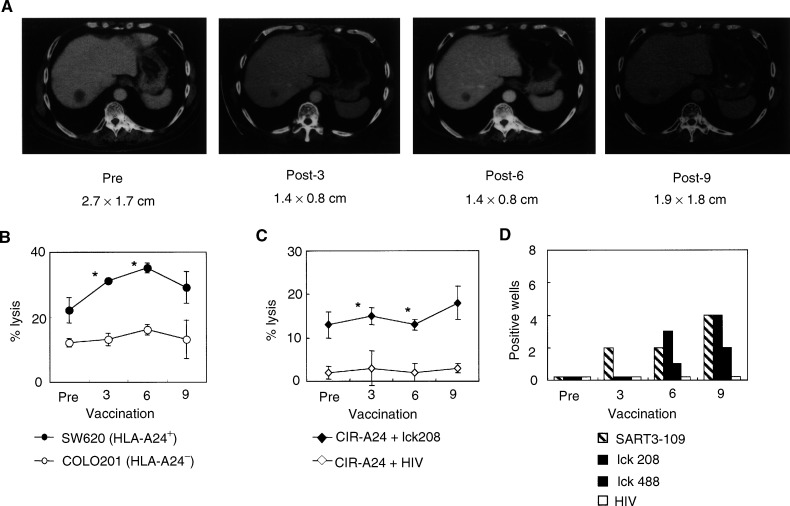
Clinical and immunological responses to the peptide vaccination. (**A**) CT scans show tumour regression of the liver metastasis after the peptide vaccination. The size of the liver metastasis (S8) is described. (**B**) CTL activity before and after vaccinations. Cytotoxicity to SW620 cells (HLA-A24^+^ colon cancer cell line), COLO201 cells (HLA-A24^−^ colon cancer cell line), and PHA-activated T cells (HLA-A24^+^) was tested by a 6-h ^51^Cr-release assay at an *E*/*T* ratio of 40/1. Values are the means of triplicate assay. ^*^Statistically significant at *P*<0.05. (**C**) Peptide-specific CTL activity before and after the peptide vaccinations. Cytotoxicity to CIR-A2402 cells preloaded with the lck_208_ or the control HIV peptide was tested by a 6-h ^51^Cr-release assay at an *E*/*T* ratio of 40/1. ^*^Statistically significant at *P*<0.05. (**D**) Kinetics of peptide-specific CTL precursors. Pre- and post- (3rd, 6th, and 9th) vaccination PBMCs were incubated at 100 cells per well in a 96-well round-microculture plate in the presence of feeder cells. The number of wells producing a significant level of IFN-*γ* in a peptide-specific manner among 96 wells is shown.

The clinical responses of each patient are summarised in Table 4. CT scans of patient 1 pre- and post-vaccination are shown in Figure 4A

. In all, 48% regression (27–14 mm) of liver metastasis was observed in patient 1. This patient had para-aortic lymph node metastasis at a diameter of less than 20 mm; it showed no change after the peptide vaccination (data not shown). Because his clinical response was considered a partial response (PR), this patient was analysed more in detail. The kinetic analysis of tumour cell lysis in patient 1 indicates that increased CTL activity to the SW620 cells became detectable in post-vaccination PBMCs (Figure 4B). The cytotoxicity against lck_208_ peptide-loaded CIR-A2402 cells became significant after the 3rd and 6th vaccinations (Figure 4C). While no peptide-specific IFN-*γ* production was detected in any of the 96 wells containing 100 cells well^−1^ of pre-vaccination PBMCs, SART3_109_-specific IFN-*γ* production was detected in two, two, and four wells among 96 wells containing post-vaccination (3rd, 6th, and 9th) PBMCs, respectively (Figure 4D). Production of lck_208_-specific IFN-*γ* production was detectable in three and four wells among 96 wells containing the 6th and 9th vaccination PBMCs, while lck_488_-specific IFN-*γ* production could be observed in one and two wells among 96 wells containing the 6th- and 9th-vaccination PBMCs, respectively. The patient has subsequently been treated only by vaccination (SART3_109_, lck_208_, and lck_488_) for 7 months as an outpatient, and is still doing well. Patient 2 had intrapelvic metastasis, and the disease has remained stable (s.d.) for 6 months. The eight other patients showed progressive disease (PD) 2–4 months after starting the vaccinations, although all have been treated as outpatients and their quality of life has been evaluated as quite high.

## DISCUSSION

Patients undergoing this regimen received 3 mg of peptides biweekly for up to four peptides. All of the peptides used were derived from nonmutated self-antigens involved in cellular proliferation (Kikuchi *et al*, 1999; Gomi *et al*, 1999; Yang *et al*, 1999; Harashima *et al*, 2000; Kawano *et al*, 2000; Nakao *et al*, 2000; Nishizaka *et al*, 2000). However, there was no grade III or IV adverse effect, which is consistent with previous observations in studies of peptide-based vaccinations. Most of the patients received the vaccination as outpatients, and the performance status remained very good throughout the treatment periods. Therefore, in terms of safety, this regimen of CTL precursor-oriented peptide vaccine could be recommended as a cancer vaccine suitable for further clinical trials.

Another aim of our study was to assess the clinical response to the vaccination. In our limited number of case, we observed one PR and one s.d. continuing for more than 6 months. Both of these cases were treated with three kinds of SART3- and lck-derived peptides, suggesting that the combined use of these peptides might constitute a promising vaccine strategy for advanced colorectal carcinomas, thus encouraging us to plan a phase II trial utilising these peptides.

Vaccination-induced immunity was evaluated in this study by several different methods, including IFN-*γ* production in response to peptides, a standard 6-h ^51^Cr-release assay, measurement of antipeptide antibody, and DTH responses. An elevated immune response to lck_208_ and lck_488_ was detected in post-vaccination PBMCs by all of the methods used in the samples of patient 1, who showed PR. This patient's PBMCs also reacted to the SART3_109_ peptide, as measured by frequency analysis of cellular responses to peptides (Figure 4D) and also by DTH test (Table 4). These results indicate that the patient's PBMCs reacted to all three vaccinated peptides after the peptide vaccination. Post-vaccination PBMCs from patient 2, who had a long s.d., responded to lck_208_ peptide alone, and the post-vaccination sera became positive for both the SART3_109_ and lck_486_ peptides, although no DTH response was observed (Table 4). Besides patient 1, positive DTH response was observed in only two patients (4 and 7), with PD, but their post-vaccination PBMCs showed no increase in cellular responses to the administered peptides. On the other hand, besides patients 1 and 2, IgG reactive to the administered peptides became detectable in the post-vaccination sera of five other patients (5, 6, 7, 9, and 10) with PD. Although the post-vaccination PBMCs of patients 6 and 10 showed an increase in cellular responses to SART3_109_ and SART3_315_, respectively, no augmentation of peptide-specific cellular response was observed in other cases. Neither a cellular nor humoral immune response to administered peptides was detectable in the remaining two patients (3 and 8), who also had PD. These results suggest that vaccination-induced immunity varies considerably among patients. However, we recently reported that the *in vivo* induction of IgG reactive to administered peptides is positively correlated with clinical response or the survival of patients with prostate, lung, gastric, or gynaecological cancer (Mine *et al*, 2003; Noguchi *et al*, 2003; Sato *et al*, 2003; Tsuda *et al*, 2004). This may be the case with patients 1 and 2, who showed PR and s.d., respectively, because IgGs reactive to two different peptides were induced only in these two patients. We have no clear answer regarding the role of peptide-specific IgG in antitumour immune response, and are now grappling with this theme.

In addressing the mechanism for peptide-specific IgG induction after peptide vaccination, one possibility is that 9-mer or 10-mer peptide-recognizing CD4^+^ T cells were involved in this phenomenon. In general, *in vivo* generation of antigen-specific IgG requires a cytokine from helper T cells (Parker, 1993). Although peptides binding to MHC class II molecules have been suggested to be 12–25 amino acids in length, the core sites anchored to MHC class II molecules are sufficient even at a length of about nine amino acids (Rammensee *et al*, 1995). Indeed, we recently observed that peptide vaccination with a 9-mer peptide could induce peptide-specific and HLA-DR-restricted CD4^+^ T cells *in vivo* (Harada *et al*, 2004). CD4^+^ T-cell help is required during the generation and maintenance of effective anti-tumour CD8^+^ T cell-mediated immunity. The requirement of CD4^+^ T-cell help to initiate and sustain a CD8^+^ T-cell response has been well established and has led to the development of antitumour vaccines that attempt to induce both T-cell subsets (Knutson *et al*, 2001). The *in vivo* induction of IgG reactive to administered peptides may be indirect evidence of the involvement of CD4^+^ T lymphocytes.

We recently developed a culture system to evaluate CTL precursors against many peptides using a limited number of PBMCs from cancer patients (Hida *et al*., 2002); the same culture system was applied to this study. The main reason why we assessed peptide-specific CTL precursors based on two parameters, the *P*-value and IFN-*γ* production, was that the levels of IFN-*γ* produced by peptide-specific CTLs varied among quadruplicate wells. This finding might be due to the small number of cells (10^5^ cells well^−1^) that were initially placed in each well. It is possible that one well may have contained peptide-specific CTL precursors, whereas another may have contained none. We concluded that each well should be individually estimated to screen for the presence of peptide-specific CTL precursors.

Recent reports revealed that a Th2 response is predominant in cancer patients (Pellegrini *et al*, 1996; Vita *et al*, 1999; Sheu *et al*, 2001). Therefore, we examined the level of IL-4 during peptide stimulation *in vitro*, but the level of IL-4 production was generally low, and no peptide-specific IL-4 production was observed. In contrast, the level of IFN-*γ* production was constantly substantial. Probably, the *in vitro* culture of PBMCs in the presence of IL-2 could preferentially activate natural killer cells, and natural killer cell-derived IFN-*γ* might provide an optimal condition for Th1 type cells.

In conclusion, vaccination of colorectal cancer patients with peptides by the CTL precursor-oriented method was a well-tolerated outpatient treatment and induced antigen-specific immunity as well as a clinical response. Even though only a small number of selected patients were treated, the encouraging clinical response demands further studies of CTL precursor-oriented vaccine in other human cancers.
